# Soluble multi−epitope protein vaccination leverages antigen availability to drive CD8^+^ T cell immunity and tumor control

**DOI:** 10.3389/fimmu.2026.1840895

**Published:** 2026-06-10

**Authors:** Thomas M. E. V. van den Brekel, Laura J. W. Goossens-Kruijssen, Katarzyna Olesek, Eleonora Nardini, Gregory M. Koningstein, Joelle van Elk, Wouter S. P. Jong, Sanne Duinkerken, S. Luirink, Yvette van Kooyk

**Affiliations:** 1 Department of Molecular Cell Biology and Immunology, Amsterdam institute for Immunology and Infectious diseases, Amsterdam UMC, Location VUmc, Amsterdam, Netherlands; 2 Amsterdam Institute for Infection and Immunity, Amsterdam, Netherlands; 3 Department of Molecular Microbiology, Amsterdam Institute of Molecular and Life Sciences, Vrije Universiteit, Amsterdam, Netherlands; 4 Abera Bioscience AB, Uppsala, Sweden

**Keywords:** adoptive transfer, cancer vaccines, *E. coli* expression system, immunotherapy, multi-epitope antigens, protein-based vaccine, therapeutic strategies

## Abstract

The shift toward precision medicine in cancer immunotherapy has increased demand for rapid, scalable production platforms for personalized vaccine antigens targeting neoantigens and tumor-associated antigens. Escherichia coli-based recombinant protein production represents a globally established system offering speed and cost-effectiveness, yet the immunogenic potential of soluble antigens produced via this platform remains incompletely characterized. Here, we systematically evaluated the capacity of E. coli-derived soluble antigen formulations to elicit anti-tumor T cell responses. Using ClearColi BL21 (DE3), a lipopolysaccharide (LPS)-truncated strain free of endotoxic contamination, we produced soluble formulations containing murine OVA-derived epitopes. *In vitro* antigen presentation assays revealed minimal T cell activation despite high numbers of CD8^+^ and CD4^+^ T cells, indicating compromised antigen presentation capacity. However, *in vivo* adoptive transfer experiments demonstrated strongly inducible T cell expansion in draining and non-draining lymph nodes, with high frequencies of antigen-specific CD8^+^ T cells producing IFN-γ and TNF-α. Prime-boost vaccination strategies in experimental melanoma models achieved protective efficacy and durable survival with persistent antigen-specific T cell responses throughout the observation period. Under stringent single-prime conditions, transient T cell frequencies were observed without long-term tumor control.

## Introduction

T cells play a central role in defending the body against intracellular infections, dysfunctional host cells, and malignant transformations such as cancer. This immune surveillance relies on the detection of antigens presented by major histocompatibility complex class I (MHC-I) molecules. While natural infections typically trigger these responses, vaccines can be designed to mimic such signals and activate antigen-specific T cells in the absence of real pathogens. In cancer immunotherapy, tumor-associated antigens, often delivered as peptides, can stimulate targeted T cell activity. However, short peptide vaccines often lack the intrinsic immunogenicity to provoke a strong response, prompting the use of carrier proteins like keyhole limpet hemocyanin (KLH) or inactivated diphtheria toxoid (DT) to boost their effectiveness (1). Other strategies include incorporating long synthetic peptides into branched polymeric structures (MAPs) or combining them with potent adjuvants to enhance immune activation (2). While these approaches aim to promote both immediate effector responses and long-term immune memory, the format in which the antigen is presented may influence how effectively these immune responses are generated.

Advancements in genomics and proteomics have revolutionized how we identify and exploit tumor- or pathogen-derived antigens for vaccine development. Techniques like high-throughput sequencing and mass spectrometry now allow researchers to pinpoint MHC-I-associated peptides presented by diseased cells. For instance, integrative approaches combining exome and transcriptome sequencing with proteomic profiling have enabled the design of personalized vaccines that elicit T cell responses specifically against cancer cells, minimizing off-target effects on healthy tissue (3–5).

While peptide-based vaccines hold promise, antigens alone are rarely sufficient to stimulate strong cellular immunity. adjuvants are essential components that act as immune activators to enhance antigen presentation and T cell priming. However, despite the success of current adjuvants in promoting antibody responses, driving strong CD8^+^ T cell activation remains a major hurdle (6, 7). Over the past decades, our understanding of dendritic cells (DCs) has deepened, particularly regarding their ability to sense danger signals and present antigens via MHC molecules. As a result, strategies that directly target DCs or modulate their activation state have emerged as key approaches for enhancing vaccine efficacy.

Importantly, while MHC-I molecules typically bind short peptides (8–10 amino acids) for CD8^+^ T cell recognition, MHC-II molecules present longer peptides (13–25 amino acids) to CD4^+^ T helper cells. Both arms of the cellular immune response play complementary roles: CD8^+^ cytotoxic T cells directly kill target cells, while CD4^+^ T helper cells provide essential support for sustaining and amplifying immune responses, including CD8^+^ T cell priming and memory formation. Delivering minimal epitopes directly can cause antigen persistence at the injection site and lead to T cell dysfunction. To circumvent this, longer peptides are preferred, as they must be processed by professional APCs, such as DCs, before presentation, thereby activating both MHC-I and MHC-II pathways and focusing the response where it is most effective. This approach has proven successful in both antiviral and cancer vaccine research (8, 9).

In *E. coli*-based recombinant protein production, maltose-binding protein (MBP) is commonly used as a fusion partner to enhance protein solubility and enable purification through amylose affinity chromatography (10, 11). Beyond these practical advantages, MBP has been shown to possess intrinsic immunogenic properties. Studies have demonstrated that MBP can activate DCs through Toll-like receptor 4 (TLR4) signaling, leading to enhanced antigen presentation and the induction of both cellular and humoral immune responses (12, 13). This positions MBP not only as a purification tag but also as a fusion partner that may contribute to enhanced vaccine immunogenicity. Importantly, by fusing both MHC-I- and MHC-II-restricted epitopes within the same recombinant protein, this approach allows simultaneous stimulation of CD8^+^ cytotoxic and CD4^+^ helper T cells, two arms of the immune system that act synergistically to generate strong and durable immune responses. A main advantage of the *E. coli* expression system is that both epitopes can be incorporated directly and as a single defined unit into one fusion construct, enabling straightforward, scalable production without the need for separate synthesis or conjugation steps. Here, we produced MBP-fused soluble antigen formulations in *E. coli* and comprehensively evaluated their capacity to generate anti-tumor T cell responses.

## Results

### Development of soluble recombinant protein antigens

To generate soluble antigen formats, constructs were designed incorporating the murine OVA-derived OT-I (CD8^+^) and OT-II (CD4^+^) epitopes (sDP-OVA, Soluble Dual Peptide-OVA), in the ClearColi BL21 (DE3) host strain. This *E. coli* strain, which produces truncated lipopolysaccharide (LPS) incapable of triggering endotoxin-mediated responses in human cells (14), eliminates inflammatory contamination that could confound immunological assessments and represents an important step toward translational applications.

**Figure 1:**
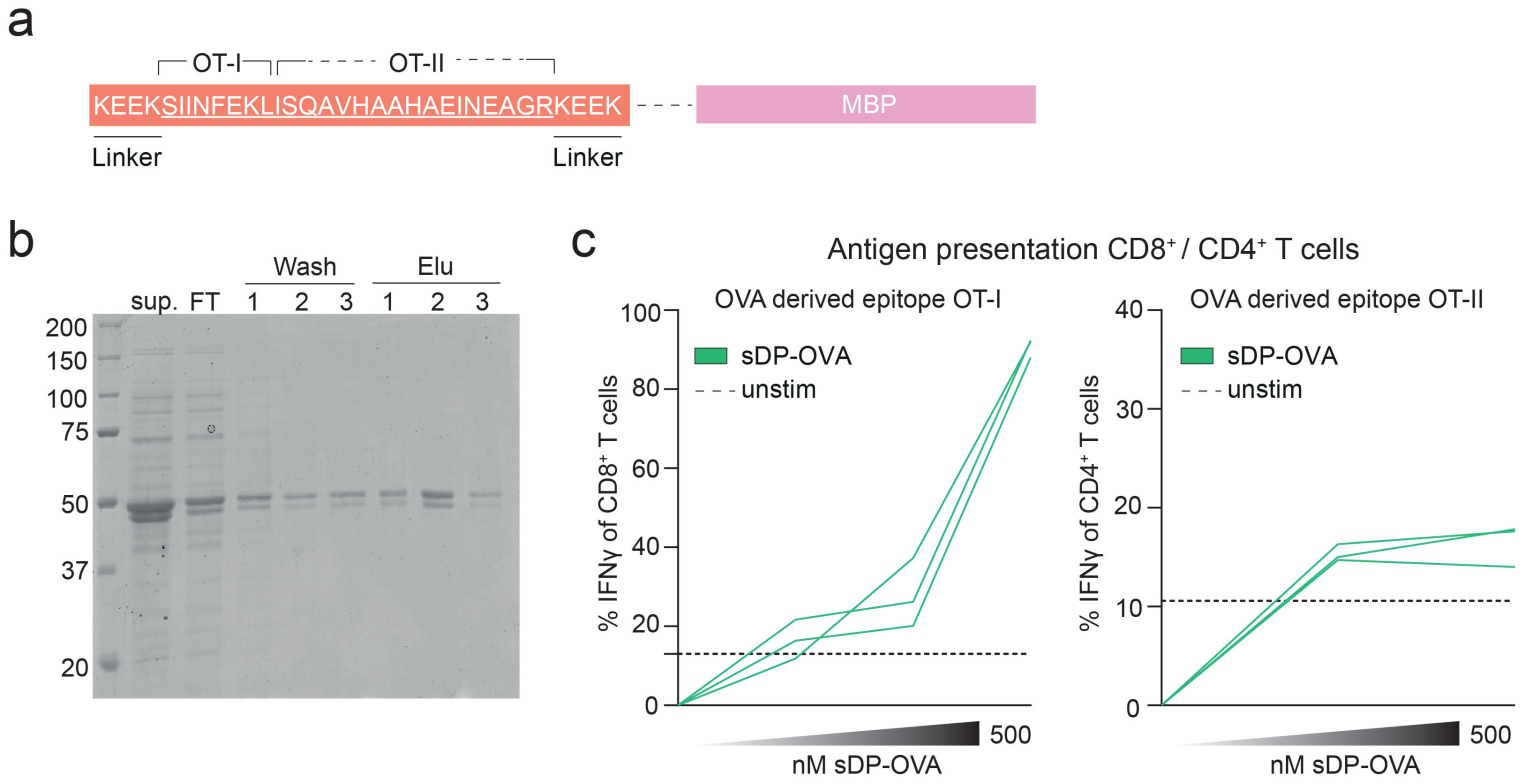
Design, purification, and antigen presentation of soluble multi-epitope protein antigens. **(a)** Recombinant OVA-derived polypeptide containing the indicated OT-I and OT-II epitopes was expressed in E. coli as an MBP fusion. **(b)** Representative SDS–PAGE analysis of the purification of the MBP-fused OVA-derived polypeptide. Lanes show molecular weight marker (ladder), soluble fraction (sup.), flow-through (FT), wash fractions (Wash 1-3), and elution fractions (Elu 1–3). **(c)** Antigen presentation was assessed in triplicate by pulsing BMDCs with sDP-OVA or leaving them unstimulated, followed by co-culture with OT-I or OT-II T cells. IFN−γ production was measured by intracellular cytokine staining after 5 h of Brefeldin A treatment.

The core design incorporates antigen sequences engineered to present MHC-I and MHC-II epitopes of interest as the primary immunogenic payload while maintaining protein solubility. To facilitate purification and characterization, we incorporated a maltose-binding protein (MBP) domain enabling streamlined purification via amylose affinity beads, a human influenza hemagglutinin (HA) tag for detection and quantification, and a SpyTag peptide to enable future coupling with other proteins possessing immunomodulatory properties (**Figure 1a**).

The production process leverages bacterial expression systems optimized for soluble protein formation and biocompatibility, yielding highly pure soluble antigens as confirmed by SDS–PAGE analysis (**Figure 1b**).

To determine whether the physical format of antigen delivery affects antigen processing and presentation by antigen-presenting cells (APCs), the capacity of soluble proteins to undergo processing and presentation by bone marrow-derived dendritic cells (BMDCs) for OVA-derived OT-I and OT-II epitopes was evaluated. This approach allowed direct assessment of the impact of sDP-OVA on dendritic cell-mediated activation of antigen-specific CD4^+^ and CD8^+^ T cell populations. BMDCs pulsed with increasing concentrations of sDP-OVA induced dose-dependent activation of OT-I CD8^+^ T cells, with IFN-γ production becoming apparent at higher antigen concentrations (**Figure 1c**). However, this response required relatively high antigen doses and remained less efficient across the concentration range compared with the long peptide control, which elicited OT-I activation at substantially lower concentrations (**Supplementary Figure 1**). In contrast, OT-II CD4^+^ T cell activation by sDP-OVA was detectable but modest and reached a plateau at lower levels, indicating limited efficiency of MHC-II presentation under these *in vitro* conditions. These findings suggest that while sDP-OVA can support CD8^+^ T cell activation when provided at sufficiently high concentrations, its intrinsic capacity for antigen uptake and/or processing by dendritic cells *in vitro* is comparatively constrained.

### *In vivo* T cell priming and kinetics of antigen-specific responses

**Figure 2:**
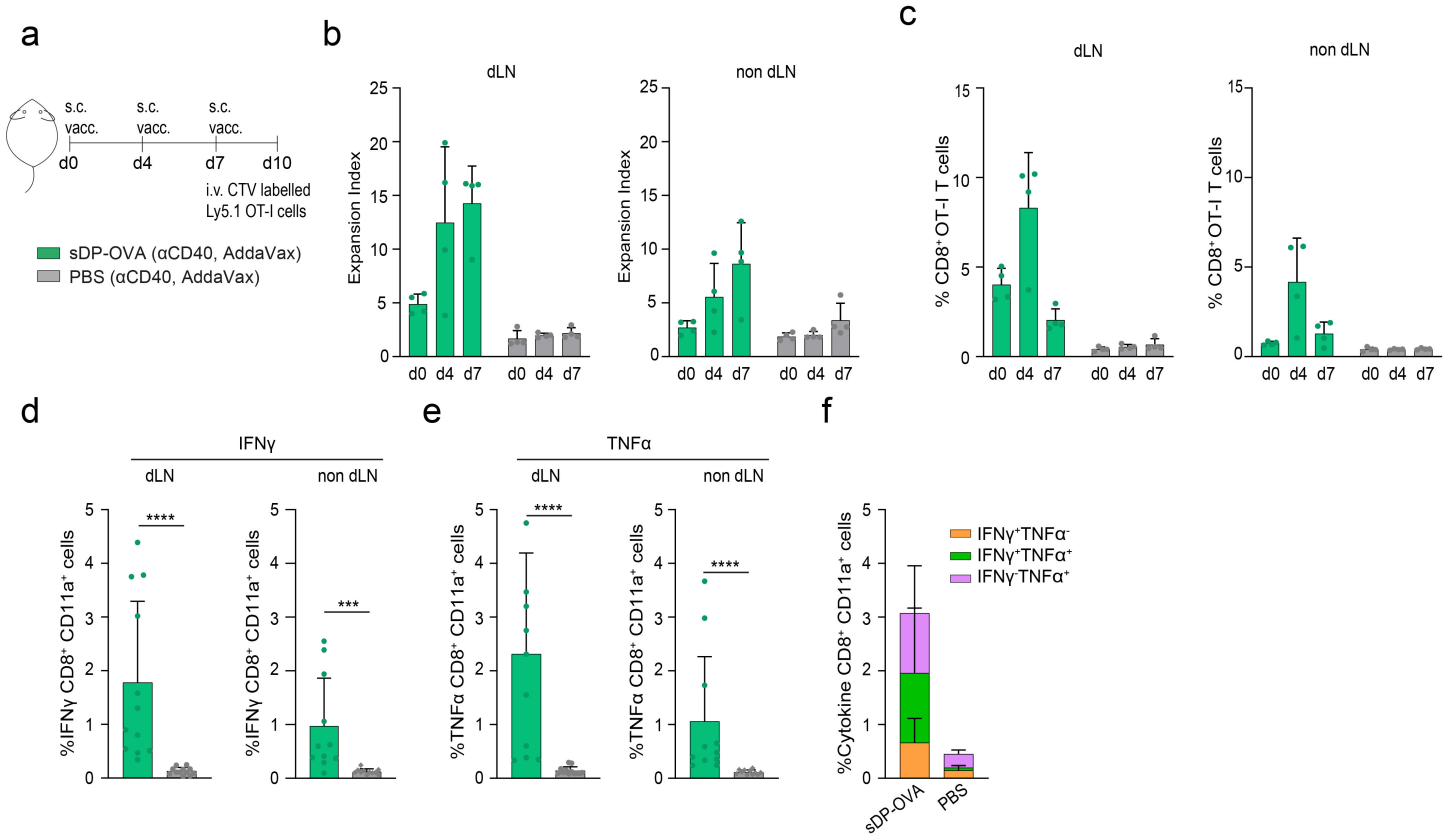
*In vivo* priming and functional activation of antigen-specific T cells following vaccination with sDP-OVA. **(a)** C57BL/6 mice were s.c. vaccinated on days 0, 4 and 7 with sDP-OVA or PBS formulated with αCD40 and AddaVax adjuvant. **(b)** CTV labelled Ly5.1 OT-I cells were injected i.v. on day 7 whilst proliferation (expansion index) was measured in both the dLN and non-dLN on day 10. **(c)** Frequencies of CD45^+^ CD3^+^ antigen-specific CD8^+^ and CD4^+^ T cells in dLN and non-dLN. **(d–f)** Single−cell suspensions from pooled dLN and non−dLN were *ex vivo* restimulated with OT−I peptide and analyzed by intracellular IFN−γ staining in gated CD8^+^ CD11a^+^ T cells to identify activated, antigen−exposed CD8^+^ T cells (left ****p< 0.0001, right ***p=0.0003, Mann-Whitney U test, n =12 biological replicates per group) or TNF-α (left ****p< 0.0001, right ****p< 0.0001, Mann-Whitney U test, n =12 biological replicates per group).

Having established weak antigen presentation profiles *in vitro*, we moved on to investigate whether the sDP-OVA formulation was able to induce antigen-specific T cell responses *in vivo*. To this end, we employed an adoptive transfer model using OT-I cells. Despite the relatively low DC maturation observed for sDP-OVA compared to the LPS control *in vitro* (**Supplementary Figure 2**), all treatment groups *in vivo* received a combination of αCD40 antibody and AddaVax adjuvant to ensure immune activation. This dual adjuvant approach was selected based on their complementary immune−stimulating properties, as αCD40 enhances DC maturation and cross−presentation, inducing efficient CD8^+^ T−cell priming (15, 16), while AddaVax, a squalene−based oil−in−water emulsion, promotes antigen retention at the injection site, recruitment of innate immune cells, and local inflammatory activation (17). To track the kinetics and magnitude of the response, C57BL/6 mice were vaccinated subcutaneously (s.c.) with sDP-OVA plus adjuvants, or adjuvants only (PBS) on day 0, 3, or 7, followed by intravenous (i.v.) transfer of CTV-labeled OT-I cells (**Figure 2a**). This staggered design allowed us to assess whether the soluble format of the antigen influences antigen persistence, uptake, or T cell priming over time, which is relevant given prior studies suggesting that soluble antigens are rapidly cleared *in vivo* (18, 19).

Interestingly, *in vivo* analysis revealed priming of adoptively transferred OT-I cells in the draining lymph node (dLN) following sDP-OVA vaccination. Analysis of OT-I proliferation demonstrated extensive cell division at all vaccination time points, as evidenced by pronounced CTV dilution (**Figure 2b**). Notably, the degree of proliferation decreased progressively from mice vaccinated on day 0 to those vaccinated on day 7, consistent with waning antigen availability or reduced duration of antigen exposure at later time points prior to OT-I transfer. Despite these differences in proliferative activity, the frequency of antigen-specific OT-I CD8^+^ T cells followed a distinct kinetic profile. Quantification of OT-I cells showed highest frequencies in the dLN of mice vaccinated on day 4, with lower frequencies observed at day 0 and day 7 (**Figure 2c**). This pattern is consistent with expected CD8^+^ T cell priming kinetics, wherein peak accumulation reflects the balance between proliferation, differentiation, and early contraction (20, 21). Importantly, both proliferation and accumulation of OT-I cells were largely restricted to the dLN, with minimal expansion detected in non-draining lymph nodes (non-dLN), indicating localized antigen uptake and presentation at the vaccination site (**Figures 2b, c**). To further assess whether the sDP-OVA delivery affected T cell functioning, we isolated lymph node cells on day 10 and performed *ex vivo* restimulation with the OT-I peptide, followed by intracellular cytokine staining. Given that IFN-γ and TNF-α are key effector cytokines involved in cytotoxic responses and tumor control (22, 23), we used these as our primary readouts. sDP-OVA-vaccinated mice generated OT-I cells that produced IFN-γ and TNF-α responses, with comparable frequencies of cytokine-positive cells (**Figures 2d, e**). Of particular interest, the proportion of polyfunctional CD8^+^ T cells, defined as cells co-producing IFN-γ and TNF-α, was elevated in the sDP-OVA-vaccinated mice compared to the PBS (adjuvants only) control mice (**Figure 2f**). This indicates that sDP-OVA not only preserved but enhanced the functional quality of the CD8^+^ T cell response, promoting a greater proportion of polyfunctional T cells, independent of the differences in antigen uptake observed *in vitro*. To determine whether these findings extended beyond the adoptive transfer setting, we also assessed endogenous immune responses following sDP-OVA immunization. Consistent with the adoptive transfer data, sDP-OVA induced detectable antigen-specific CD8^+^ T cell responses and effector cytokine production, whereas CD4^+^ T cell responses remained minimal (**Supplementary Figures 3a–c**). In addition to cellular immune responses, prime-boost immunization with sDP-OVA resulted in a detectable serum anti-OVA antibody response (total Ig levels) (**Supplementary Figure 3d**). These results demonstrate that sDP-OVA is capable of eliciting functional T cell immunity *in vivo* when delivered s.c.

### *In vivo* antitumor efficacy and persistence of vaccine-induced T cell responses

**Figure 3:**
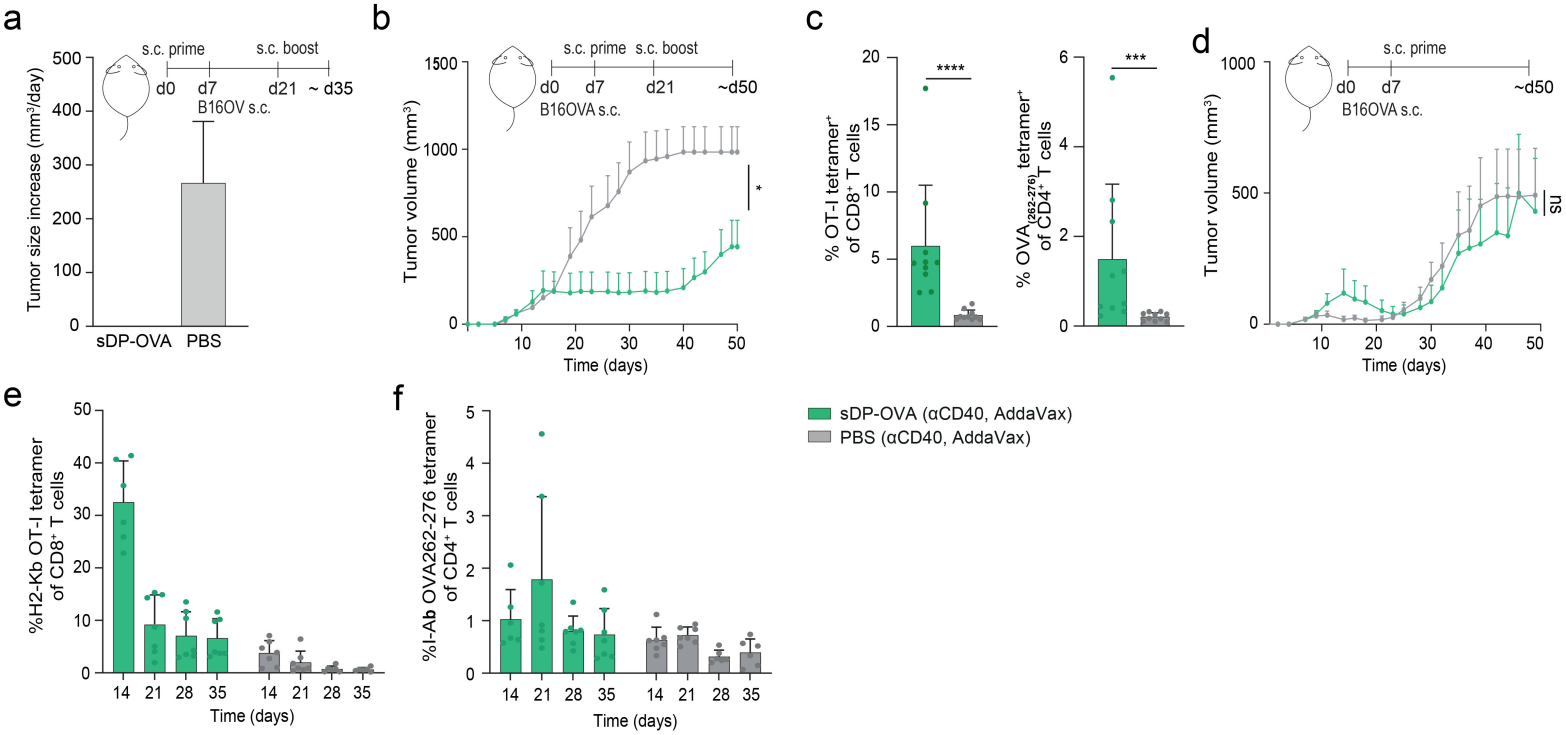
Efficacy of antigen-specific sDP-OVA vaccine in B16-OVA models. **(a)** Tumor size increase (mm^3^/day) of prophylactic s.c. treatment on day 7 and s.c. inoculation of 300.000 B16-OVA cells on day 0 (****p < 0.0001, Mann-Whitney U test, n =10 biological replicates per group). **(b)** Tumor growth curves of mice receiving s.c. inoculation of 300.000 B16-OVA cells on day 0 and therapeutic s.c. vaccination on day 7 (and day 21) when tumor was palpable (*p= 0.0186, Mann-Whitney U test, n =10 biological replicates per group). **(c)** Comparison of antigen-specific T cells in blood on day 14. Left: H-2Kb OT-I tetramer staining (****p< 0.0001, Mann-Whitney U test, n = 10 biological replicates per group) and right: I-Ab OVA_262–276_ tetramer staining (***p = 0.0002, Mann-Whitney U test, n = 10 biological replicates per group). **(d)** Tumor growth curves of mice receiving s.c. inoculation of 300.000 B16-OVA cells on day 0 and a single dose therapeutic s.c. vaccination on day 7 when tumor was palpable (ns = 0.7302, Mann-Whitney U test, n = 10 biological replicates per group). **(e, f)** Comparison of antigen-specific T cells in blood on day 14, 21, 25, and 35.

Having established that sDP-OVA elicited T cell responses in the adoptive transfer model, we next sought to determine whether this would translate into protective efficacy against tumor challenge. To address this question, we designed an *in vivo* prophylactic model in which antigen exposure preceded tumor establishment, allowing us to evaluate the protective capacity of vaccine-primed immune responses in a clinically relevant prevention scenario. Mice were vaccinated s.c. with either sDP-OVA or PBS, both adjuvanted with αCD40 and AddaVax, before s.c. inoculation of B16-OVA tumor cells in the flank. Interestingly, all sDP-OVA vaccinated mice showed complete protection against tumor establishment, with no measurable tumor growth observed throughout the monitoring period. In contrast, adjuvant-only control mice showed progressive tumor growth and succumbed within 28 days (**Figure 3a**). To study the therapeutic potential of sDP-OVA, we used the same melanoma tumor model in which tumor-bearing B16-OVA mice were treated with the same prime-boost vaccination protocol as in the prophylactic study, and tumor growth was monitored over an extended period. Mice vaccinated with sDP-OVA initially controlled tumor growth up to day 40 (**Figure 3b**). However, beyond this time point, tumor progression was observed. Notably, in contrast to the prophylactic setting, where prime-boost vaccination conferred complete protection and sustained tumor remission for the full duration of the study, the therapeutic setting demonstrated only partial efficacy. To explore whether differences in antigen persistence could underlie the observed long-term protection, we analyzed circulating antigen-specific T cell responses following vaccination. Analysis of peripheral blood at day 7 post-vaccination (day 14 post–tumor inoculation) revealed markedly increased frequencies of antigen-specific CD8^+^ T cells, as measured by OVA_257-264_ (H-2K^b^) tetramer staining, in sDP-OVA-vaccinated mice compared with adjuvant-only controls (**Figure 3c**). In parallel, elevated frequencies of OVA_262–276_ specific CD4^+^ T cells were detected using I-A^b^ tetramers. Given that soluble antigens exhibit rapid clearance kinetics from injection sites and circulation (24, 25), we next examined whether reducing antigen exposure to a single vaccination would alter the functional activity of the soluble formulation. In the absence of a booster vaccination, tumor growth in the sDP-OVA vaccine group was initially delayed compared to the adjuvant-only control, indicating successful T cell priming and early immune activation. However, this protection waned over time, with tumor growth beginning to accelerate rapidly after day 30, ultimately resulting in widespread outgrowth in all animals in this group (**Figure 3d**). Tracking tetramer-positive CD8^+^ and CD4^+^ T cells in peripheral blood over time revealed a transient peak around day 14 (7 days post-vaccination) in sDP-OVA vaccinated mice, after which antigen-specific T cell frequencies declined (**Figures 3e, f**). Together, these data indicate that sDP−OVA induces antigen−specific T cell responses that are sufficient for prophylactic tumor protection but are insufficient to sustain long−term control in the therapeutic setting.

## Discussion

We show that our soluble multi-epitope protein antigen sDP-OVA elicited antigen-specific CD8^+^ and CD4^+^ T cell responses *in vivo*, provided complete protection in a prophylactic tumor model, and mediated transient tumor control in a therapeutic setting, despite limited DC maturation and lower antigen presentation than the positive control *in vitro*. This apparent discrepancy between standard *in vitro* readouts and *in vivo* vaccine performance is well documented in the literature. Classical DC activation markers and peptide-MHC display only partially reflect the complex tissue, kinetic, and inflammatory cues that govern productive T cell priming and antitumor immunity *in vivo* (26–28).

Using an adoptive transfer setup in which vaccination and T cell transfer were separated by different time intervals, we found that sDP-OVA supported CD8^+^ T cell priming *in vivo*. Adoptively transferred OT-I cells proliferated strongly and accumulated in dLN, but not in non-dLN, in line with models in which antigen uptake after s.c. vaccination and naive T cell priming are largely restricted to draining lymphoid tissues (29, 30). As the gap between vaccination and T cell transfer increased, OT-I proliferation declined, indicating that sDP-OVA-derived antigen remains available for several days, but that the strength of the response depends on the timing of antigen encounter (31, 32). In line with this, antigen-specific CD8^+^ T cells displayed expected priming kinetics, with peak frequencies in mice vaccinated 3 days prior to OT-I transfer and reduced frequencies when vaccination occurred 7 days earlier, consistent with canonical expansion, effector differentiation, and early contraction as antigen availability diminishes (20, 21). Together, these findings indicate that *in vivo* priming dynamics are regulated predominantly by antigen availability and tissue context, rather than by antigen presentation efficiency measured *in vitro*.

Beyond proliferation, sDP−OVA−primed CD8^+^ T cells remained functional, with IFN−γ and TNF−α co−production upon *ex vivo* restimulation, a polyfunctional profile that has been linked to stronger protective and antitumor responses in infection and cancer immunotherapy (33, 34). Conventional prime-boost immunization elicited detectable endogenous antigen-specific CD8^+^ T cell responses and effector cytokine production, showing that sDP-OVA can drive functional cellular immunity outside the adoptive transfer setting, in line with recent protein- and peptide-based cancer vaccine studies (35, 36).

These T cell responses translated into tumor control *in vivo*. In prophylactic vaccination models, sDP−OVA completely prevented tumor outgrowth when antigen exposure preceded tumor challenge, consistent with preclinical and early clinical studies in which vaccination before tumor establishment or surgery conferred strong protection (35, 36). In therapeutic models, sDP-OVA vaccination initially slowed tumor growth, but this effect was lost over time, especially when only a single priming dose was given. This pattern suggests that sDP-OVA can initiate antitumor immunity, but that long-term control of established tumors depends on continued immune pressure, which a short antigen pulse may not support (31, 32). The shorter timeframe in the adoptive transfer and prophylactic studies may also have obscured late differences in vaccine performance. Prior work shows that small soluble proteins clear quickly via lymphatics, shortening antigen presentation and limiting repeated T cell activation (37–40). In many models, this is overcome by repeated dosing or by combining vaccination with checkpoint blockade to sustain effector T cells in the tumor microenvironment (26, 41, 42).

Prime-boost immunization with sDP-OVA also induced detectable serum anti-OVA antibodies. This was observed despite low OT-II CD4^+^ T cell responses in our assays, suggesting that antibody production may be supported by helper mechanisms that we did not capture, such as low-frequency CD4^+^ T cells or extrafollicular B cell responses. Adjuvanted protein vaccines are known to drive class-switched antibodies via germinal center and extrafollicular routes, giving rise to long-lived plasma cells and memory B cells (43). However, recent work highlights that extrafollicular and other non-germinal center B cell responses can also generate class-switched antibodies under conditions of limited T cell help or strong innate stimulation, providing a plausible explanation for antibody production in the absence of strong OT-II expansion (44). The ability of soluble recombinant proteins to induce antibody responses may reflect their accessibility to B cell receptors and triggering of humoral immune pathways. Although the functional contribution of antibodies to tumor control was not directly assessed in this study, their role in cancer vaccination remains an area of ongoing debate, with context-dependent evidence for both beneficial and limited contributions. Recent analyses of cancer vaccines and nanovaccine platforms highlight that vaccine-induced antibodies can contribute to antitumor immunity through several mechanisms, including direct tumor targeting, Fc-mediated effector functions, and promotion of antigen spreading. At the same time, these studies emphasize that the relative contribution of humoral responses to durable tumor control can vary considerably between antigens, vaccine platforms, and tumor types (45). Thus, in our model, antigen-specific serum Ig documents humoral activation, but its net effect on tumor control remains to be tested.

To improve therapeutic performance, several optimization strategies can be envisioned for this platform. One approach is to alter the physical format of sDP−OVA from a rapidly cleared soluble protein to particulate or depot−forming formulations, such as liposomes, polymeric or inorganic nanoparticles, protein assemblies, or functional inclusion bodies. These strategies have been shown to enhance antigen retention in dLN and potentiate T−cell priming in preclinical cancer vaccine models using comparable antigen systems (46, 47). Nanovaccines and related particulate delivery systems can increase co−delivery of antigen and adjuvant, enable multivalent antigen display, and prolong antigen availability, all of which have been linked to more potent CD8^+^ T−cell responses and improved tumor control compared with soluble vaccines (47). In addition, lymph node–targeted delivery strategies, including size−optimized nanoparticles, biomaterial depots positioned in or near dLNs, and bacterial inclusion−body depots that provide slow local release of therapeutic proteins, have been shown to increase antigen and adjuvant accumulation in draining (including tumor−draining) LNs (48–52). These approaches can thereby enhance the efficacy of therapeutic vaccination in tumor models. Adapting sDP-OVA to such particulate, inclusion-body–based, or lymph−node–targeted formats, in combination with rational adjuvant selection and dosing or with immune checkpoint blockade, may therefore provide a route to translate the strong prophylactic activity observed here into more durable therapeutic benefit.

Overall, our findings support a model in which the immunogenic potential of soluble protein antigens is shaped by antigen availability, anatomical localization, and adjuvant support *in vivo*, rather than by *in vitro* presentation efficiency alone. When paired with strong adjuvants and suitable dosing regimens, soluble antigens can prime CD8^+^ T cells, maintain effector function, and support antitumor activity. Studies using adjuvanted protein and peptide vaccines, including mRNA-boosted and nanovaccine formulations, show that extending antigen persistence or improving delivery to lymphoid tissues can enhance CD8^+^ T cell responses and tumor control compared with rapidly cleared soluble antigens (43, 45). Prior work also indicates that co-delivery of antigen and adjuvant to the same draining lymph node and multivalent antigen presentation further strengthen T cell priming and tumor control (45). The impact of antigen availability on response longevity is therefore shaped by the spatial and temporal context of antigen presentation, which can be modulated by adjuvant selection, antigen multimerization, and the anatomical localization of vaccine components *in vivo*. Our data are consistent with these concepts and suggest that both the site of antigen presentation in draining lymph nodes and the time window of antigen availability influence the size and durability of protein vaccine-induced cellular immunity.

## Materials and methods

### Cell lines

B16-OVA melanoma cells (gift from Prof. T.N. Schumacher, Netherlands Cancer Institute) (53) were grown in RPMI-1640 medium (Gibco, Life Technologies) supplemented with 10% FCS (Biowest), 50 U/ml penicillin, 50 μg/ml streptomycin, and 2 mM L-glutamine (all from Lonza). Cells were cultured at 37 °C in a humidified atmosphere containing 5% CO_2_ and were routinely passaged and collected for experiments at approximately 70% confluency.

### Strains and plasmids

Plasmid constructs were generated using a combination of restriction enzyme–based cloning and In-Fusion HD Cloning Kit–mediated assembly. Each plasmid encoded antigenic peptide inserts containing defined MHC-I and MHC-II epitopes. Maltose-binding protein (MBP) was incorporated as a purification handle, an HA tag was included to facilitate additional purification or detection, and a SpyTag (version 3) motif was appended to enable covalent coupling to immunomodulatory proteins. Model ovalbumin (OVA) epitopes comprised the CD8^+^ T cell epitope OVA257-264 (SIINFEKL; H-2K^b^-restricted OT-I epitope) together with the CD4^+^ T cell epitope OVA323-339 (ISQAVHAAHAEINEAGR; OT-II epitope), which were synthesized as Geneblocks (IDT) and arranged either in tandem or separated by defined linker sequences (KEEK SIINFEKL ISQAVHAAHAEINEAGRKEE) as indicated. Because transgenic OT-II CD4^+^ T cells were used throughout for *in vitro* assays and *in vivo* immunizations (53, 54), constructs incorporating both OT-I and OT-II epitopes were prepared. In wild-type C57BL/6 mice, however, the dominant CD4^+^ T cell determinant is OVA262-276 (EKLTEWTSSNVMEER) (55); therefore, additional soluble recombinant proteins containing OT-I together with the OVA262-276 epitope were produced and used in all *in vivo* tumor experiments. These two epitopes are contiguous within the native OVA protein, so flanking residues were maintained to generate the following inserted sequence: DEVSGLEQLESIINFEKLTEWTSSNVMEERKIK. All final constructs were verified by Sanger sequencing (Macrogen, Amsterdam, The Netherlands).

### Production of sDP-OVA

Endotoxin-free E. coli strain ClearColi BL21(DE3) harboring the OTIOTII-MBP-HA-SpT3 and (OTI(OVA262-276))-MBP-HA-SpT3 derived plasmids were induced for protein expression with anhydrotetracycline (0.2 mg/ml, 50% EDTA). After collection by centrifugation (6,000x g, 10 min, 4oC), cells were resuspended in column buffer (20 mM Tris-HCl pH 7.4, 0.2 M NaCl, 1 mM EDTA) at a concentration of 50 OD/mL. Protease inhibitor PMSF was added to a final concentration of 1 mM immediately prior to cell disruption. Cells were lysed by two passes through a high-pressure cell disruptor (One-Shot, 1.2 kbar). Insoluble material was removed by centrifugation (10,000 × g, 10 min, 4 °C), followed by ultracentrifugation of the supernatant (293,000 × g, 1 h, 4 °C) to obtain a clarified lysate. The cleared lysate was incubated with amylose resin (New England Biolabs) for 1 h at room temperature followed by 1 h at 4 °C under gentle agitation. After binding, the resin was washed extensively with column buffer, and bound proteins were eluted with column buffer supplemented with 10 mM maltose. Protein expression and purification efficiency were assessed by SDS-PAGE analysis. Selected elution fractions were pooled and dialyzed against phosphate-buffered saline (PBS) using regenerated cellulose dialysis tubing with a molecular weight cut-off of 3.5 kDa (Spectra/Por). Dialysis was performed at 4 °C under gentle stirring with at least one buffer exchange, after which dialyzed protein was recovered and stored at −20 °C. Samples were analyzed on 10% SDS-PAGE gels, stained with Coomassie Blue G250 (Biorad, CA, USA). To allow protein quantification, gels were scanned using a Molecular Imager GS800 Calibrated Densitometer (Bulletin, Life Science, USA) and the intensities of protein bands were determined using ImageJ (http://rsb.info.nih.gov/ij/). Concentrations of samples was quantified from Coomassie stained gels containing BSA reference standards.

### *In vitro* antigen presentation

BMDCs from C57BL/6 mice were differentiated from bone marrow cells cultured in 5 cm non-treated Petri dishes in IMDM (Gibco, Thermo Fisher) supplemented with 10% FCS (Biowest), 50 U/ml penicillin, 50 μg/ml streptomycin, 2 mM L-glutamine, and 50 μM β-mercaptoethanol (all from Lonza), together with 40 ng/ml GM-CSF (ImmunoTools; hereafter referred to as BMDC medium). Fresh BMDC medium containing GM-CSF was added on day 2 of culture. On days 7-8, plates were rinsed with PBS (Fresenius Kabi), and loosely adherent and non-adherent cells were harvested, pelleted at 300 g for 5 min, resuspended in BMDC medium at 5 × 10^5^ cells/ml, and seeded into 96-well plates. BMDCs were incubated with sDP−OVA at 5, 50, or 500 nM (0.25, 2.5, or 25 µg/mL) or with long peptide OVA (KEEKSIINFEKLISQAVHAAHAEINEAGRKEEC) at 4.7, 47, or 470 nM (0.25, 2.5, or 25 µg/mL) for 3 h at 37 °C. After incubation, plates were centrifuged (300 g, 5 min), supernatants were aspirated, and cells were washed three times. Spleens from naïve OT-I and OT-II C57BL/6 mice were collected, passed through 100 μm strainers, and washed in RPMI-1640 (Gibco, Life Technologies) containing 10% FCS (Biowest), 50 U/ml penicillin, 50 μg/ml streptomycin, and 2 mM L-glutamine (all Lonza). Red blood cells were lysed using ACK buffer (Gibco, Thermo Fisher), followed by dilution and washing in complete RPMI. OT-I and OT-II T cells were enriched from splenocyte suspensions using the MagniSort™ Mouse T Cell Enrichment Kits (CD4: 8804-6821-74; CD8: 8804-6822-74; Thermo Fisher), according to the manufacturer’s instructions. Purified T cells were resuspended in T cell medium (complete RPMI supplemented with 50 μM β-mercaptoethanol) at 1 × 10^6^ cells/ml and labeled with CellTrace Violet (CTV; Thermo Fisher) at a 1:2000 dilution for 7 min at 37 °C, then washed three times. Labeled OT-I/OT-II cells were resuspended in BMDC medium at 5 × 10^4^ cells/well and added to the BMDC cultures. Co-cultures were maintained at 37 °C in a humidified 5% CO_2_ incubator. On day 3, 5 μg/ml Brefeldin A (ER-Golgi transport inhibitor; InvivoGen) was added together with 0.1 μg/ml phorbol 12-myristate 13-acetate (PMA; Thermo Fisher) and 0.5 μg/ml ionomycin (Thermo Fisher) for 5 h, after which cells were processed for flow cytometric analysis of T cell proliferation and intracellular cytokine (IFN-γ and TNF-α) production.

### Animal experiments

All procedures involving animals were conducted under approval of the institutional Animal Welfare Body (IvD, CCD protocol AVD11400202216545). C57BL/6 mice (equal numbers of males and females) were obtained at 4 weeks of age from Charles River and allowed to acclimatize for 4 additional weeks at the Animal Research Institute Amsterdam (ARIA). Animals were maintained at Amsterdam UMC in accordance with institutional and national regulations, housed under a 12 h light-dark cycle with ad libitum access to food and water. Group sizes were based on prior experience and statistical power considerations, and animals were randomly assigned to experimental groups.

### Immunization *in vivo*

Mice were immunized s.c. in the neck on day 0 with 2 nmol sDP−OVA (circa 100 µg protein per mouse; MW 50.6 kDa) of the indicated formulations, together with 25 µg in−house–produced αCD40 antibody, formulated 1:1 (v/v) with AddaVax (InvivoGen; 50% final volume). A booster dose was administered s.c. on day 14, and spleens were collected on day 21. Spleens were placed in RPMI-complete medium (RPMI-1640; Gibco, Life Technologies) supplemented with 10% FCS (Biowest), 50 U/ml penicillin, 50 μg/ml streptomycin, and 2 mM L-glutamine (all Lonza), then gently dissociated through pre-wetted 100 μm cell strainers. Cell suspensions were centrifuged (1500 rpm, 6 min), treated with ACK red blood cell lysis buffer (Gibco), and washed in complete RPMI. After counting, splenocytes were allocated either for direct tetramer staining and full-spectrum flow cytometry or for *ex vivo* restimulation assays.

### *Ex vivo* restimulation

Splenocytes were divided over two culture plates for CD8^+^ and CD4^+^ T cell analyses and restimulated under epitope-specific conditions. For CD8^+^ T cell (OT-I) restimulation, cells were cultured with 0.1 μg/ml OT-I peptide (SIINFEKL) in the presence of 5 μg/ml Brefeldin A (ER–Golgi protein transport inhibitor; InvivoGen) for 5 h at 37 °C. These cultures were subsequently processed for assessment of T cell proliferation and intracellular cytokine (IFN-γ and TNF-α) production. For CD4^+^ T cell (OT-II) restimulation, splenocytes were incubated overnight at 37 °C with 10 μg/ml OT-II peptide (ISQAVHAAHAEINEAGR). The following day, 5 μg/ml Brefeldin A was added for an additional 5 h incubation at 37 °C, after which cells were stained for full-spectrum flow cytometry as detailed in **Table 1**. In all experiments, stimulation with 0.1 μg/ml phorbol 12-myristate 13-acetate (PMA; Thermo Fisher) plus 0.5 μg/ml ionomycin (Thermo Fisher) was included as a positive control for T cell activation.

**Table 1:** Antibody list for full spectrum flow cytometry.

Marker	Fluorchrome	Clone	Company	Panel
CD8b	PE	H35-17.2	Invitrogen	Antigen presentation
CD4	PE	GK1.5	Invitrogen	Antigen presentation
IFN- γ	APC	XMG1.2	Invitrogen	Antigen presentation, *ex vivo* restimulation
FVD	ef780	–	eBioscience™	Antigen presentation, Adoptive transfer *in vivo*, *ex vivo* restimulation, B16-OVA *in vivo’s*
CD16/32	–	93	eBioscience™	Adoptive transfer *in vivo*, B16-OVA *in vivo’s*
CD8a	AF700	53-6.7	Biolegend	Adoptive transfer *in vivo, ex vivo* restimulation
OT-I tet	PE	H-2Kb/SIINFEKL	NIH Tetramer Core Facility	Adoptive transfer *in vivo*, B16-OVA *in vivo’s*
OVA262–276 tet	APC	I-A/I-E/EKLTEWTSSNVMEER	NIH Tetramer Core Facility	Adoptive transfer *in vivo*, B16-OVA *in vivo’s*
CD3	BUV395	17A2	BD Biosciences	Adoptive transfer *in vivo*, B16-OVA *in vivo’s*
CD4	BUV563	GK1.5	BD Biosciences	Adoptive transfer *in vivo*, B16-OVA *in vivo’s*
CD3e	PE-Cy5	145-2C11	Biolegend	*ex vivo* restimulation
CD11a	FITC	M17/4	Biolegend	*ex vivo* restimulation
TNF-α	PE-Cy7	MAb11	Biolegend	*ex vivo* restimulation
CD45	Pacific Blue	30-F11	Biolegend	B16-OVA *in vivo’s*

### Adoptive transfer *in vivo*

C57BL/6 (CD45.2^+^) mice were immunized s.c. in the neck with sDP−OVA (2 nmol, circa 100 µg protein per dose; MW 50.6 kDa) or PBS (negative control) on days 0, 4, and 7, with all formulations containing 25 µg in−house–produced αCD40 and mixed 1:1 (v/v) with AddaVax (InvivoGen). On day 7 after the first vaccination, mice received an intravenous transfer of 3 × 10^5^ OT−I CD8^+^ T cells isolated from Ly5.1 (CD45.1^+^) OT−I transgenic mice (kind gift from the group of Prof. D. Amsen, Sanquin, Amsterdam, the Netherlands). OT−I CD8^+^ T cells were purified from spleen and lymph nodes using a negative−selection MagniSort™ Mouse CD8^+^ T cell Enrichment Kit (Thermo Fisher Scientific) following the manufacturer’s protocol, starting from single−cell suspensions prepared by mechanical dissociation and ACK red blood cell lysis. Enriched CD8^+^ T cells were labeled with CellTrace Violet (CTV; Thermo Fisher Scientific) at 1 µM in PBS with 2% FCS for 20 min at 37 °C in the dark, after which staining was quenched with excess complete RPMI, cells were washed, resuspended in RPMI−complete medium, and injected intravenously into recipient mice. Three days after adoptive transfer, mice were sacrificed and draining and non−draining lymph nodes were collected. Lymph nodes were mechanically disrupted and digested for 30 min at 37 °C in RPMI containing Liberase TL (100 µg/ml) and DNase I (50 µg/ml) with gentle agitation. Digestion was stopped with ice−cold RPMI supplemented with 10% FCS, 10 mM EDTA, 20 mM HEPES, and 20 µM 2−mercaptoethanol, followed by further dissociation by pipetting, filtration through a 70 µm strainer, and washing to obtain single−cell suspensions. Cells were then divided for immediate *ex vivo* phenotypic staining and functional analysis. One fraction was stained with a comprehensive T−cell antibody panel including markers for donor identification (CD45.1), antigen specificity, proliferation (CTV dilution), and effector/memory subsets and analyzed by full−spectrum flow cytometry. The remaining cells were restimulated *ex vivo* with OT−I peptide, followed by intracellular cytokine staining for IFN−γ and TNF−α and acquisition on the same full−spectrum cytometer using standard gating strategies.

### B16-OVA s.c. tumor *in vivo* experiments

For tumor challenge, 3 × 10^5^ B16-OVA cells in 100 µl sterile PBS were injected s.c. into the flank. Tumor growth was monitored every 1–3 days using caliper measurements to calculate tumor volume with a standard ellipsoid-based formula: $\left(\frac{4}{3}\right)\pi \left(\frac{length}{2}\right)\left(\frac{width}{2}\right)\left(\frac{average}{2}\right)$. In the prophylactic regimen, mice received s.c. vaccinations in the neck with 2 nmol antigen (approximately 100 µg protein per mouse) on days 0 and 21, each formulated with 25 μg in-house–produced αCD40 and mixed 1:1 with AddaVax (InvivoGen), followed by s.c. tumor inoculation in the flank on day 7. For the prime-boost therapeutic protocol, tumors were first allowed to establish for 7 days before administering a 2 nmol vaccine dose (plus 25 μg αCD40 and 50% AddaVax) s.c. in the neck, with a booster given on day 21. In the single-prime therapeutic setting, tumor-bearing mice similarly received a single 2 nmol vaccination (again supplemented with 25 μg αCD40 and 50% AddaVax) 7 days after tumor implantation. Animals were euthanized when tumors exceeded 800 mm³, when body weight loss surpassed 20%, or when other humane endpoints defined by veterinary staff were met. Peripheral blood was collected either by cheek puncture or sampling from the vena saphena, as judged appropriate by veterinary personnel, into heparinized tubes. Subsequently, 0.5 ml of pre-warmed (37 °C) ACK lysis buffer was added, samples were incubated at room temperature, and cells were washed. If red blood cells persisted (indicated by a visible red pellet), an additional 100 µl ACK buffer was applied for 2 min, followed by neutralization with 100 µl PBS and centrifugation at 300 g for 6 min at 2-8 °C. The resulting pellet was washed once more, counted, and plated for downstream flow cytometric staining.

### Flow cytometry

Cells were first incubated for 15 min at 4 °C with 20 µg/ml anti-CD16/32 (clone 2.4G2, in-house produced) together with Fixable Viability Dye eFluor 780 (1:1000; eBioscience). After washing, antigen-specific T cells were identified by staining with H-2K^b^/SIINFEKL tetramers for CD8^+^ T cells and I-A/I-E tetramers loaded with ISQAVHAAHAEINEAGRKEE (for *in vitro* and immunization experiments) or EKLTEWTSSNVMEER (for *in vivo* tumor studies) for 45 min at 37 °C. Cells were then labeled with a surface antibody panel (**Table 1**) for 30 min at 4 °C, fixed in 2% paraformaldehyde (Electron Microscopy Sciences) for 15 min at 4 °C, washed three times, and acquired on a Cytek Aurora 4L full-spectrum cytometer (16UV-16V-14B-8R). Data was analyzed using SpectroFlo and Omiq softwares. For *ex vivo* restimulation assays, cells were first stained with the same surface antibody panel (**Table 1**) for 30 min at 4 °C, then fixed in 2% paraformaldehyde as above. Fixed cells were washed and permeabilized in 0.5% saponin (Sigma) containing intracellular staining antibodies (**Table 1**) for 30 min at 4 °C, washed again, and analyzed on the Cytek Aurora 4L using the same instrument configuration.

### ELISA for OVA-specific total Ig in serum

Unless otherwise specified, all incubation steps were performed at room temperature. High-binding Maxisorp plates were coated overnight at 4 °C with 50 µl per well of OVA (5 µg/ml; Sigma) diluted in sodium phosphate coating buffer (11.8 g Na_2_HPO_4_ and 16.1 g NaH_2_PO_4_ dissolved in 1 L Milli-Q water, pH 6.5). Plates were washed three times with PBS containing 0.05% Tween-20, then blocked with 200 µl/well of 1% BSA in PBS for 1 h. Serum samples were diluted in 1% BSA/PBS, and 50 µl of each dilution was added to the coated, blocked wells and incubated for 2 h. After three washes with PBS/0.05% Tween-20, bound antibodies were detected with polyclonal rabbit anti-mouse Ig-HRP (Dako P0161) diluted 1:2000 in 1% BSA/PBS (50 µl/well, 1 h incubation). Plates were then washed five times with PBS containing 0.05% Tween-20. For color development, TMB substrate was freshly prepared by diluting TMB stock solution (10 mg/ml in DMSO) 1:100 in 0.11 M sodium acetate buffer (NaAc; pH 5.5) and adding 1 µl of 30% H_2_O_2_ per 10 ml substrate solution, and 50 µl was dispensed per well. Plates were incubated protected from light until a clear blue signal developed, after which the reaction was stopped with 100 µl of 2 N H_2_SO_4_ per well and absorbance was read at 450 nm.

### Statistics

Statistical analyses and P value calculations were performed using GraphPad Prism versions 9 and 10. The specific statistical tests used for each experiment are detailed in the corresponding figure legends. For comparisons involving two groups, normality was assessed using the Shapiro-Wilk test, but as data were consistently non-normally distributed, a Mann-Whitney U test was applied in all cases. For comparisons involving three groups, normality was assessed using the Shapiro-Wilk test. All data normality was not met (P< 0.05), so a Kruskal-Wallis test was applied, followed by Dunn’s multiple comparison test. All statistical tests were two-tailed, with P > 0.05 considered not significant (NS).

## Data Availability

The original contributions presented in the study are included in the article/**Supplementary Material**. Further inquiries can be directed to the corresponding author.

## Supplementary material

Supplementary Figure 1 Antigen presentation of soluble multi-epitope protein antigens Antigen presentation was assessed by pulsing BMDCs with sDP-OVA, long peptide OVA or leaving them unstimulated, followed by co-culture with OT-I or OT-II T cells. IFN-γ production was measured by intracellular cytokine staining after 5 h of Brefeldin A treatment.

Supplementary Figure 2 sDP-OVA does not induce phenotypic maturation of BMDCs **a** BMDCs were incubated with sDP-OVA at the indicated concentrations (125, 25, or 2.5 µg/mL), lipopolysaccharide (LPS) as a positive control, or left unstimulated. After 3 h of stimulation, all conditions were extensively washed and cultures were maintained overnight. The next day, surface expression of the costimulatory molecules CD86 **(a)** and CD70 **(b)**, as well as MHC class I **(c)** and MHC class II **(d)**, was assessed by flow cytometry and quantified as mean fluorescence intensity (MFI).

Supplementary Figure 3 Endogenous immune responses following sDP-OVA immunization **(a)** C57BL/6 mice were s.c. vaccinated on day 0 and day 21 with sDP-OVA, longpeptide OVA or PBS formulated with aCD40 and AddaVax adjuvant, and sacrifsed on day 28. Frequencies of antigen-specific CD8^+^ T cells in splenocytes were analyzed (****p< 0.0001, Kruskal Wallis with Dunn’s multiple comparison test, n =8 biological replicates per group). **(b, c)** Single cell suspensions of slenocytes were pooled and *ex vivo* restimulated with OT-I short peptide and stained intracellular for cytokines IFN-γ or TNF-α (left **p=0.0014 and *p=0.0497, right **p=0.0016 Kruskal Wallis with Dunn’s multiple comparison test, n =8 biological replicates per group). **(d)** Anti-OVA total Ig response in serum from immunized mice on day 21.
