# Supplementary figures and images for: Soluble multi−epitope protein vaccination leverages antigen availability to drive CD8+ T cell immunity and tumor control
Source: Front Immunol. 2026 Jun 10;17:1840895. doi: 10.3389/fimmu.2026.1840895 (PMC13290799; doi:10.3389/fimmu.2026.1840895)

# Antigen presentation CD8<sup>+</sup> / CD4<sup>+</sup> T cells

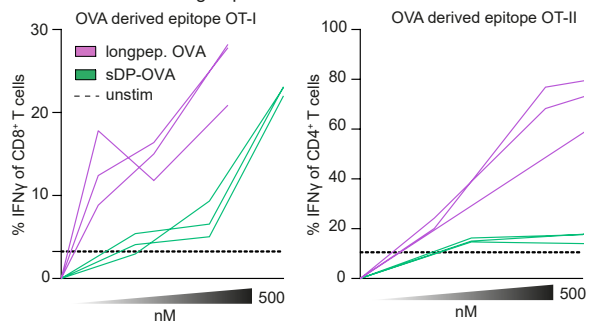

Supplement: Supplementary Figure 1 — Antigen presentation of soluble multi-epitope protein antigens Antigen presentation was assessed by pulsing BMDCs with sDP-OVA, long peptide OVA or leaving them unstimulated, followed by co-culture with OT-I or OT-II T cells. IFN-γ production was measured by intracellular cytokine staining after 5 h of Brefeldin A treatment. [file SupplementaryFile1.pdf]

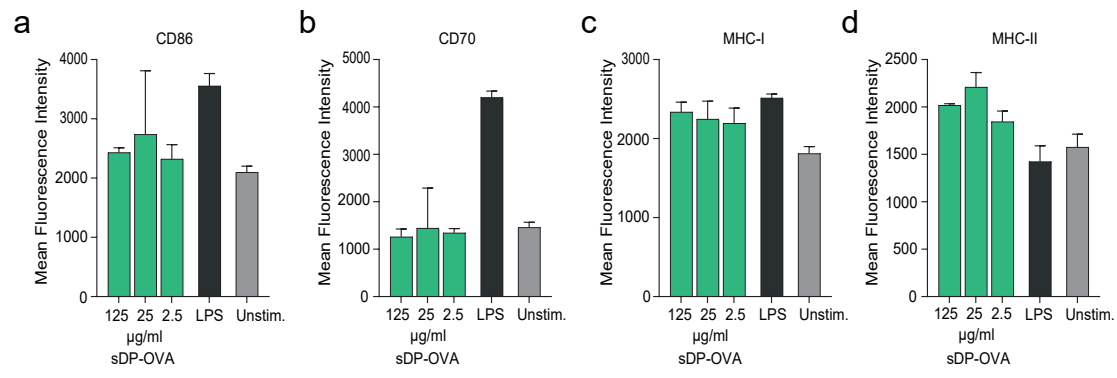

Supplement: Supplementary Figure 2 — sDP-OVA does not induce phenotypic maturation of BMDCs a BMDCs were incubated with sDP-OVA at the indicated concentrations (125, 25, or 2.5 µg/mL), lipopolysaccharide (LPS) as a positive control, or left unstimulated. After 3 h of stimulation, all conditions were extensively washed and cultures were maintained overnight. The next day, surface expression of the costimulatory molecules CD86 (a) and CD70 (b), as well as MHC class I (c) and MHC class II (d), was assessed by flow cytometry and quantified as mean fluorescence intensity (MFI). [file SupplementaryFile2.pdf]

a

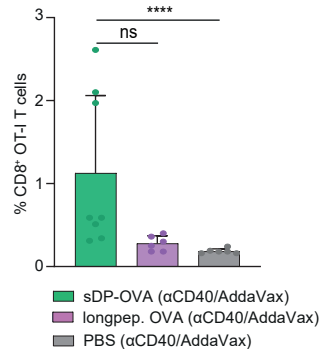

b

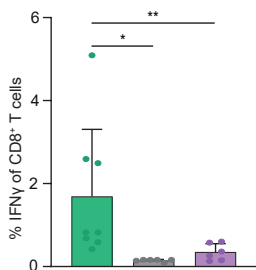

c

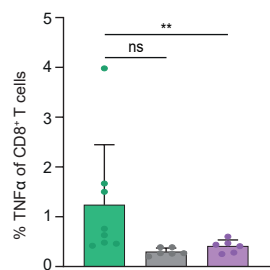

d

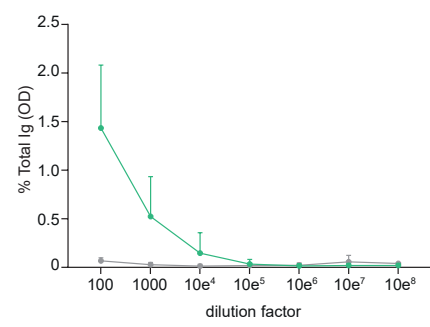

Supplement: Supplementary Figure 3 — Endogenous immune responses following sDP-OVA immunization (a) C57BL/6 mice were s.c. vaccinated on day 0 and day 21 with sDP-OVA, longpeptide OVA or PBS formulated with aCD40 and AddaVax adjuvant, and sacrifsed on day 28. Frequencies of antigen-specific CD8+ T cells in splenocytes were analyzed (****p< 0.0001, Kruskal Wallis with Dunn’s multiple comparison test, n =8 biological replicates per group). (b, c) Single cell suspensions of slenocytes were pooled and ex vivo restimulated with OT-I short peptide and stained intracellular for cytokines IFN-γ or TNF-α (left **p=0.0014 and *p=0.0497, right **p=0.0016 Kruskal Wallis with Dunn’s multiple comparison test, n =8 biological replicates per group). (d) Anti-OVA total Ig response in serum from immunized mice on day 21. [file SupplementaryFile3.pdf]
